# Much obesity, scarce staff: the inequality of human resources in nutrition in Mexico (2019-2023)

**DOI:** 10.17843/rpmesp.2026.433.16389

**Published:** 2026-09-30

**Authors:** Elizabeth Hoyos-Loya, Jair Yañez-Santaolalla, María Cecilia González-Robledo, David A. Barrera-Núñez, Mónica Ancira-Moreno, Karla María Armendáriz-Acosta, Hortensia Reyes-Morales, Jaqueline E. Alcalde Rabanal

**Affiliations:** 1 Centro de Investigación en Sistemas de Salud, Instituto Nacional de Salud Pública, Cuernavaca, Morelos, Mexico. Instituto Nacional de Salud Pública Centro de Investigación en Sistemas de Salud Instituto Nacional de Salud Pública Cuernavaca Morelos Mexico; 2 Centro de Investigación en Salud Poblacional, Instituto Nacional de Salud Pública, Cuernavaca, Morelos, Mexico. Instituto Nacional de Salud Pública Centro de Investigación en Salud Poblacional Instituto Nacional de Salud Pública Cuernavaca Morelos Mexico; 3 Observatorio Materno Infantil, Departamento de Salud, Universidad Iberoamericana, Ciudad de México, Mexico. Universidad Iberoamericana Ciudad de México Observatorio Materno Infantil Departamento de Salud Universidad Iberoamericana Ciudad de México Mexico; 4 Departamento de Ciencias de la Salud, Universidad Autónoma de Ciudad Juárez, Ciudad Juárez, Chihuahua, Mexico. Universidad Autónoma de Ciudad Juárez Departamento de Ciencias de la Salud Universidad Autónoma de Ciudad Juárez Ciudad Juárez Chihuahua Mexico

**Keywords:** Workforce, Nutrition Personnel, Health Services, Health Inequality Monitoring, Health Care Levels, Obesity

## Abstract

**Objectives.:**

To analyze the density and inequality of human resources in nutrition (HRN) in the Mexican public health system, as well as their evolution between 2019 and 2023.

**Materials and methods.:**

An ecological study was conducted with secondary information sources for 2019 and 2023. The density of HRN per 100,000 inhabitants was estimated, based on the total population, and its distribution was analyzed according to social security institution, level of care, and federative entity. In addition, the density of HRN for adults with overweight or obesity (OoO) was estimated for 2023. To estimate the inequality in the state distribution of HRN, the concentration index (CI) was used, and the marginalization index as a stratifier.

**Results.:**

In Mexico, the density of HRN increased from 5.9 in 2019 to 6.9 per 100,000 inhabitants in 2023; however, this increase was unequal among federative entities, institutions, and levels of care. In second and third-level care units, the density reached up to 22.41 HRN per 100,000 inhabitants, while in the first level it was 2.0. There is a higher concentration of HRN in entities with lower marginalization (CI=0.13 in 2019; CI=0.11 in 2023), units for the population with social security (CI=0.36 in 2019; CI=0.33 in 2023), and second and third levels (CI=0.20 in 2019; CI=0.18 in 2023).

**Conclusions.:**

In Mexico, between 2019 and 2023, the density of HRN increased, but state-level and by level of care inequalities persist. Strengthening its planning and distribution, particularly at the first level of care, is essential to respond to the nutritional and epidemiological needs of the country.

## INTRODUCTION

A health system that guarantees care for its entire population requires having trained health personnel and in sufficient quantity to ensure the effective and quality provision of services ^1^. However, in practice, the availability and distribution of human resources for health (HRH) continues to be heterogeneous worldwide, with differences between countries and regions that can limit or compromise the response capacity of health systems ^1^^,^^2^. This problem is compounded by the HRH shortage projected by the World Health Organization (WHO), where a deficit of 11 million professionals is projected for the year 2030, which will particularly affect middle and low-income countries, including Mexico ^3^.

Given this scenario, during the last two decades, WHO and other international organizations have incorporated the strengthening of HRH into the global agenda, with emphasis on their availability and accessibility. Among the most relevant efforts is the Global Strategy on Human Resources for Health published in 2016, which emphasizes the need for sufficient HRH, adapted to local conditions, and aligned with the current and future health needs of populations ^(^^1^.

These recommendations acquire special importance in countries like Mexico, which during the last decades, has experienced a transformation in its epidemiological profile. The different forms of malnutrition —both due to excess and due to nutrient deficiency— have consolidated as an important public health problem and a challenge for the health system, highlighting the high prevalences of overweight or obesity (OoO) ^4^. According to the National Health and Nutrition Survey (ENSANUT) (2020-2023), 36.5% of schoolchildren, 40.4% of adolescents and 75.2% of adults live with OoO ^(^^5^^,^^6^. Furthermore, 29.9% of the adult population has a diagnosis of hypertension and 18.3% of diabetes, where only one in three adults has metabolic control of the disease ^7^^,^^8^. The complications derived from these conditions are among the main causes of death in the country ^9^.

Obesity should be recognized and treated as a chronic and complex disease, determined by the interaction of biological, behavioral, and social factors ^10^. Its care requires a comprehensive and interdisciplinary care model, supported by multidisciplinary health teams—including professionals in medicine, nursing, psychology, and nutrition—among others, with sufficient capacities for comprehensive and organized diagnosis, treatment, and follow-up. Evidence shows that these models can improve cardiometabolic outcomes and the quality of life of people living with this condition ^11^^,^^12^.

In this context, although the promotion of healthy habits and basic counseling can be performed by different members of the healthcare team, human resources in nutrition (HRN) play a fundamental role, and provide the specific competencies to perform nutritional assessment and diagnosis, design and implement individualized intervention plans, prescribe nutritional treatment, and monitor the results of interventions in people with OoO and their associated comorbidities ^13^^,^^14^. Furthermore, HRN participates in the design, implementation, and evaluation of healthy eating strategies and programs at the individual and population levels. These clinical and public health competencies distinguish specialized nutritional care from general counseling and support the need to incorporate them into health services ^15^. Their integration into health services has been associated with improved health outcomes and with the strengthening of the capacities of other HRH for the comprehensive approach to OoO as well as other chronic conditions ^16^.

However, the availability and distribution of HRH are conditioned by the organization and structure of health systems. In Mexico, the health system is highly segmented and fragmented; the population with formal employment has access to social security institutions (Mexican Social Security Institute -IMSS-, Institute of Security and Social Services for State Workers -ISSSTE-, Mexican Petroleums -PEMEX-, Ministry of National Defense -SEDENA-, and Ministry of the Navy -SEMAR-), while people with informal employment —including unemployed individuals, homemakers, and other population groups— can access State Health Services and the Decentralized Public Organism Named Health Services of the Mexican Social Security Institute for Well-being (OPD-IMSS-Bienestar), which includes care in health centers up to National Institutes, where specialized care is provided. Each institution has differentiated provision and financing guidelines, which affects the heterogeneity of the service provision model and the assignment of functions for HRH according to the institution in which they work. Consequently, the priority for hiring, availability, and distribution of HRH also differs among institutions ^17^.

Despite the importance of HRN, their availability in the Mexican health system has been poorly documented. Therefore, the objective of this study was to analyze the density and inequality of HRN in the Mexican public health system, as well as their evolution between 2019 and 2023.

**KEY MESSAGES**

Motivation for the study. Human resources in nutrition (HRN) play a fundamental role in the prevention and care of diseases; however, their availability and distribution in Mexico’s public health system have been little studied.

Main findings. Between 2019 and 2023, the national density of HRN increased from 5.97 to 6.91 per 100,000 inhabitants. However, its growth was uneven, concentrating in entities with less marginalization, second and third level units, and services aimed at the population with social security.

Public health implications. Mexico requires a federal public policy, coordinated with primary healthcare, that promotes the distribution, reorganization, and sustainable incorporation of HRN into the public health system.

## MATERIALS AND METHODS

An ecological, exploratory, multi-group study was carried out, with a temporal comparison between the years 2019 and 2023.

### Information sources

Secondary databases and administrative records were used to estimate the size of HRN, the population, and adults with OoO. For HRN, open-access information from the Sectoral Health Resources database of the General Directorate of Health Information (DGIS) was used, which contains national-level information on the characteristics of health units and available human resources ^18^. A data cleaning process was followed that allowed for the identification of records corresponding to the study universe. Details by levels of care and type of institution are detailed in the section on numerator construction.

For the national population size, the DGIS Population database was used, which presents mid-year projections of the population registry by age group, social security status, and federal entity until the year 2070 ^19^.

For the calculation of adult individuals (≥19 years) with OoO at national and state level, we used information from the ENSANUT Continua from the 2020-2024 period, which assesses the health and nutrition status of the Mexican population and which, as a whole, has representativeness at the state level ^20^.

### HRN Density

Following the framework proposed by WHO/UNICEF for the assessment of HRN availability in the general population, the research team developed and operationalized six of the seven indicators used in this study (table 1)^16^^,^^21^^,^^22^. All densities were estimated at national and state level per 100,000 inhabitants for the period 2019 to 2023, with the exception of HRN density for adults with OoO, which was calculated only for 2023. This was due to limitations in the state representativeness of adult population estimates with OoO for each of the years in the 2020-2023 period, therefore, the available information from ENSANUT Continua 2020-2024 was used. In 2019, a specific ENSANUT survey that allowed estimating the adult population with OoO was not conducted.

**Table 1:**
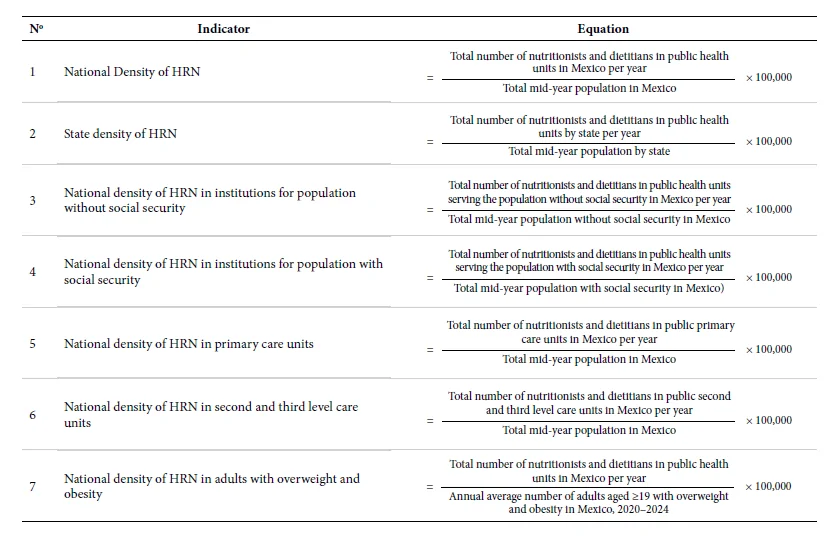
Equations for the estimation of human resources densities in nutrition.

HRN: human resources in nutrition.

*Construction of the numerators*

Registered nutritionists/dietitians in public health institutions that offer services in outpatient and hospital units were included as HRN ^16^. Nutrition personnel working in administrative support units, offices, laboratories, social assistance units, and addiction prevention centers were excluded because they do not provide nutritional services.

For the HRN in health units that serve the population without social security, the personnel assigned to units of the State Health Services, OPD IMSS-Bienestar, and the IMSS-Bienestar program were considered. In contrast, the HRH in public units intended for the population with social security incorporated professionals from IMSS, ISSSTE —including their state units—, PEMEX, SEDENA, and SEMAR.

Regarding the level of care, the HRN were classified according to the unit category reported in the database. First-level care units (FLC) were considered those classified as “outpatient consultation” which include health centers, family medicine units, medical specialty units, and mobile units. In the second and third level of care, professionals assigned to units classified as “hospital” were included, corresponding to general, regional, and specialty hospitals, as well as national health institutes ^22^.

*Construction of the denominators*

The reference population corresponded to the total estimated mid-year population for all ages, at the national and state levels. This same information was also used to make estimates according to social security status.

By level of care, it was considered that the entire population can be a potential user of the FLC given that anyone may need these services. However, for the potential second and third level population, it was assumed that the population that might require hospital or specialist care in one year is 22.0% ^23^.

Additionally, to estimate the adult population with OoO, a database with information from ENSANUT Continua 2020-2024 was integrated. These surveys were grouped to have state-level representativeness of the indicator of interest ^24^. For this purpose, participants with complete weight and height data were identified. With this information, the body mass index (BMI) was calculated, classifying as the overweight population those who presented average BMI values between 25.0 and 29.9 kg/m^2^, and as the obese population those who registered values equal to or greater than 30.0 kg/m^2^. Implausible values were excluded, defined as BMI <10.0 and >58.0 kg/m^2 (^^5^. To obtain the denominator corresponding to the adult population with OoO, absolute population sizes were estimated using the expansion factors of ENSANUT Continua 2020-2024. Since multiple survey rounds were grouped, the original expansion factors were adjusted by dividing them by the number of available rounds per federal entity (five cycles for most states, and four for Sinaloa due to lack of data in 2024). This adjustment allowed for obtaining an annualized weighted value. Considering the survey design variables, the total average annual population with OoO for each state was estimated. To incorporate the sampling variability of the survey, 95% confidence intervals (95% CI) were calculated for the state population totals. Thus, the upper limit of the density was calculated using the lower limit of the population size confidence interval as the denominator, while the lower limit of the density was estimated using the upper limit of the population size confidence interval.

To evaluate the changes in densities between 2019 and 2023, the percentage of change between both years was calculated. Likewise, a two-tailed Student’s t-test was used to evaluate the statistical significance of the observed differences. A p-value of <0.05 was considered statistically significant.

*Inequality analysis*

To quantify the inequality in the distribution of HRN among institutions for population with and without social security, as well as among levels of care and federal entity, density ratio estimators were used. This metric allows comparing the relative frequency of the indicators, providing a simple measure of the differences in their distribution ^25^.

Inequality in the distribution of HRN among the 32 federal entities was estimated using the concentration index (CI) ^26^, using the state marginalization index (MI), estimated by the National Population Council (CONAPO), as an equity stratifier. The MI is a multidimensional indicator that evidences the socio-spatial inequities present among the states of the country. Its calculation is based on nine indicators grouped into four dimensions: education, housing conditions, monetary income, and population distribution. Because this index is estimated every five years, for the present analysis, the 2020 MI ^27^ was used. The formula for estimating the CI is the following ^26^:


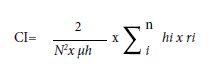


The CI result ranges from -1 and 1. A negative CI indicates that HRN density is concentrated among the most marginalized states (very high MI). Whereas, positive CIs indicate that the density is concentrated in the least marginalized states (very low MI). If the CI takes a value of zero, it indicates absence of inequality ^26^. To determine if the CI differences between both years are statistically significant, we performed a two-tailed Student’s t-test for each indicator. p-values <0.05 were considered significant.

The concentration curves were generated for each of the indicators. Which shows the graphical representation of inequality, by relating the socioeconomic dimension -MI- (X-axis) with the health resource indicator -HRN- (Y-axis); in the total absence of inequality, the curve takes the form of a straight line with a 45-degree inclination, which is called the line of equality (CI=0). The area below or above this 45-degree line corresponds to the value of the CI. Data processing was performed using the statistical package Stata version 17.0 (Stata Statistical Software, 2017; StataCorp, College Station, TX).

## RESULTS

Between 2019 and 2023, the national density of HRN increased from 5.97 to 6.91 per 100,000 inhabitants, representing an increase in the period of 15.75%. Meanwhile, for the population with OoO in 2023, the national density reached 14.09 HRN per 100,000 adults with both conditions (95% CI 13.66‒14.54). Regarding HRN in institutions for the population with and without social security, the densities were similar; however, the largest increase was recorded in institutions for the population with social security, with an increase of 25.8% (p<0.05) going from 5.62 HRN in 2019 to 7.07 in 2023; meanwhile, in institutions for the population without social security, there was an increase of 8.13% going from 6.27 in 2019 to 6.78 in 2023.

When analyzing by level of care, the highest concentration of resources was observed in second and third-level units, with 20.09 and 22.41 HRN per 100,000 inhabitants in 2019 and 2023, respectively. However, the largest increase by level of care was recorded in FLC units, with 27.74% (p<0.05). However, these units showed the lowest HRN rates: 1.55 in 2019 and 1.98 in 2023. The density ratio between the FLC and second and third-level units went from 12.96 in 2019 to 11.31 in 2023, which is equivalent to one HRN in FLC for every 11 in second and third-level units (table 2).

**Table 2:** Changes in the availability of human resources in nutrition per 100,000 inhabitants in Mexico, 2019 and 2023.

Category		2019			2023			% of change	p-value^a^
		n	Density	Density ratio	n	Density	Density ratio		
National		7569	5.97	NA	9070	6.91	NA	15.75	<0.001
National in adults with overweight and obesity		NA	NA	NA	9070	14.09 (13.66-14.54)^b^	NA	NA	NA
Social security									
	Institutions for population without social security	4333	6.27	Ref.^a^	4825	6.78	Ref.	8.13	0.140
	Institutions for population with social security	3236	5.62	0.90	4245	7.07	1.04	25.80	<0.001
By level of care									
	First-level units	1974	1.55	Ref.	2599	1.98	Ref.	27.74	0.021
	Units of second and third level	5595	20.09	12.96	6471	22.41	11.31	11.54	<0.001
Total population		126 577 891			131 230 255			3.67	

a Two-tailed Student’s t-test. A p value <0.05 was considered statistically significant, ^b^ 95% confidence interval.

Ref: reference for the estimation of the density ratio, NA: information not available.

Upon disaggregating by federal entity, in 2023, 40.62% of the entities (n=13) presented densities higher than the national average (6.91 per 100,000 inhabitants). The highest densities were observed in Mexico City (15.58), Tabasco (13.61), and Yucatán (11.19), while the lowest correspond to the states of Puebla (4.42), Oaxaca (4.02), and San Luis Potosí (3.59). The analysis of the percentage of change throughout the period evidences considerable variability among the federal entities; 65.62% of the entities (n=21) registered increases higher than the national average (15.75%), with increases that reached up to 89.22% in Hidalgo and 75.44% in Sinaloa. In contrast, 15.62% of the entities (n=5) showed reductions in the density of HRN, highlighting Tlaxcala, with a decrease of 33.91%, and the State of Mexico, with 21.15% (figure 1).

**Figure 1:**
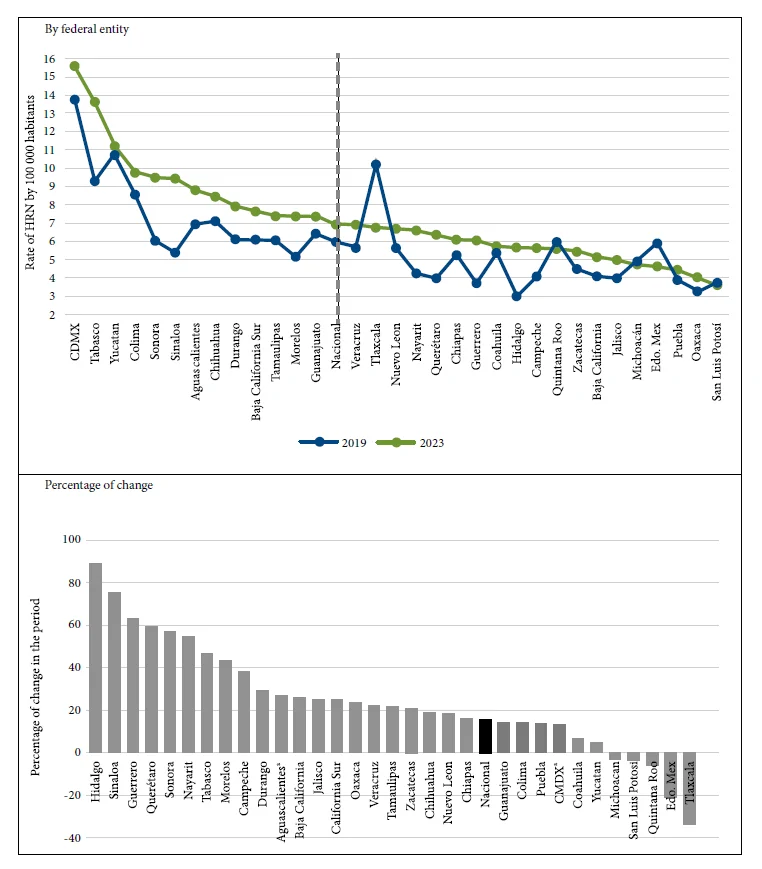
Density and percentage change in the rate of human resources in nutrition per 100,000 inhabitants in Mexico, 2019 and 2023.

^a^ Two-tailed Student’s t-test. A p-value of <0.05 was considered statistically significant. For Mexico City (CDMX) the p-value was <0.001.

The CI revealed that the highest density of HRN is concentrated in entities with lower degrees of marginalization (MI: very low), with positive and statistically significant values for the years 2019 and 2023 (CI=0.13 in 2019, p<0.05; CI=0.11 in 2023, p<0.05). When disaggregating the CI by type of public health institution, a greater, statistically significant, inequality was observed in the state distribution of HRN in institutions serving the population with social security (CI=0.36 in 2019, p<0.05; CI=0.33 in 2023, p<0.05), compared to institutions serving the population without social security. When analyzing the CI by level of care, inequality was more pronounced in second and third level units (CI=0.20 in 2019, p<0.05; CI=0.18 in 2023, p<0.05). In FLC units, the CI presented negative values close to zero in 2019 (CI=−0.05) and 2023 (CI=−0.02) (table 3). Although the percentages of change indicate reductions in the CI between 2019 and 2023 in the indicators, these differences were not statistically significant.

**Table 3:** Concentration index of human resources in nutrition according to state marginalization degree. Mexico, 2019 and 2023.

		2019			2023			% change	p-value^b^
		CI	p-value^a^	SE	CI	p-value^a^	SE		
National		0.13	<0.001	0.04	0.11	<0.001	0.04	-15.38	0.725
Adults with overweight or obesity		NA	NA	NA	0.06	0.076	0.05	NA	NA
	Social security								
	Institutions for population without social security	0.14	0.073	0.07	0.08	0.161	0.06	-42.86	0.518
	Institutions for population with social security	0.36	<0.001	0.07	0.33	<0.001	0.07	-8.33	0.763
By level of care									
	Primary Care Units	-0.05	0.352	0.05	-0.02	0.553	0.04	-60.00	0.641
	Units of Second and third level	0.20	<0.001	0.04	0.18	<0.001	0.04	-10.00	0.725

N=32

a Wald test for CI. A p-value <0.05 was considered statistically significant.

b Two-tailed Student’s t-test. A p-value <0.05 was considered statistically significant.

CI: concentration index, SE: standard error, NA: no data available.

The concentration curves for HRN density at the national level, in adults with OoO, by type of social security, as well as by level of care, were consistent with the results of the CI, by being located below the line of equity, evidencing a higher concentration of HRN in entities with less marginalization. On the other hand, the curve corresponding to the FLC units should be interpreted with caution, because it presents crossings with the line of equity, which could reflect the coexistence of inequalities of opposite magnitude and direction along the distribution of marginalization (figure 2).

**Figure 2:**
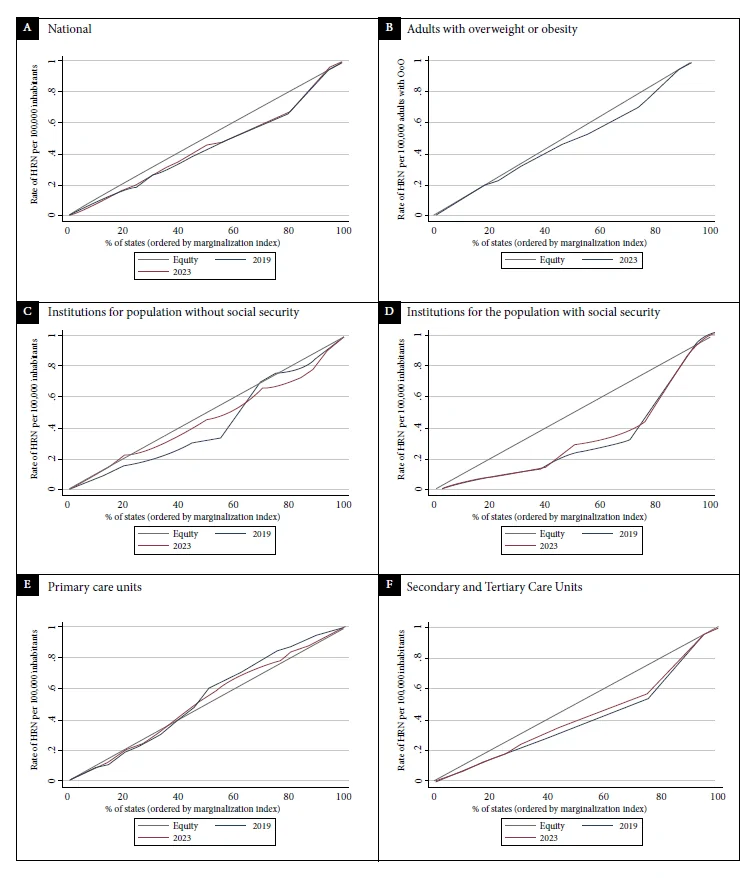
Concentration curves of human resources in nutrition according to state marginalization degree. Mexico, 2019 and 2023.

N=32 HRN: human resources in nutrition

## DISCUSSION

To our knowledge, this is the first study to document the availability and distribution of HRN in Mexico’s public health institutions. Between 2019 and 2023, the national density of these professionals increased from 5.97 to 6.91 per 100,000 inhabitants. However, this expansion was unequal among federal entities, institutions, and levels of care. HRN continued to concentrate in social security institutions and second- and third-level care units (22.41 HRN per 100,000 inhabitants in 2023), while FLC units registered a density of 1.98 professionals per 100,000 inhabitants during the same year. Positive CIs show that HRN were primarily located in entities with a lower degree of marginalization. This distribution could limit the system’s capacity to carry out actions of prevention, timely detection, and comprehensive care for multiple problems related to OoO, as well as non-communicable chronic diseases (NCDs) in outpatient contexts.

The moderate increase in the national density of HRN should not, therefore, be interpreted as evidence that Mexico has resolved its workforce gap. Firstly, there is no national or international standard that establishes a minimum or reference density of HRN necessary to respond to the needs of the population (disease burden and population size), which makes it difficult to quantify the gaps. Therefore, it is necessary to establish an indispensable minimum or contextualized planning parameters that allow guaranteeing adequate coverage, considering that their function goes beyond the prescription of diets in the hospital setting and that their participation is particularly relevant in the FLC to promote healthy nutrition. Secondly, the observed increase in HRN has been marked by temporary hiring and their incorporation into specific programs, rather than a sustained strategy for RHR planning ^28^. Finally, this density must be interpreted in light of the epidemiological context, characterized by a high prevalence of OoO from early life stages, as well as diabetes and hypertension that continue to present insufficient control ^(^^7^^,^^8^.

Our findings are consistent with evidence from other health systems in Latin America. In Brazil, the quantitative increase of these professionals has not by itself resolved geographic disparities ^29^, while in our study, a similar concentration of HRN is observed in regions of lower marginalization. Likewise, there is a critical asymmetry by levels of care. While in Peru, 7.93% of FLC establishments have a nutritionist ^30^, in Mexico, in 2023, there was one nutrition professional in the FLC for every 11 located in higher complexity units. Unlike Peru ^30^^,^^31^, our study incorporates two dimensions not explored in the Peruvian analysis: the distribution according to social security status and the formal estimation of inequalities using the state CI ^30^.

The evidence is strong in pointing out the multiple benefits to population health and health systems by integrating this resource into their care teams ^(32-34)^. These benefits range from improvements in clinical indicators ^33^, such as lipid profile, glycemic profile, BMI, and blood pressure, to the reduction of healthcare costs through lower utilization of health services, which also contributes to decreasing the saturation of hospital services and healthcare expenditure ^(32-34)^. Together, these findings demonstrate that investing in HRN, mainly in primary care, could constitute a cost-effective strategy to strengthen the healthcare response ^32^^-^^35^.

Furthermore, the study also identifies a marked gap in the distribution of HRN between second and third level hospitals and FLC units, consistent with what has been observed in Brazil and Peru ^(29-31)^. This concentration is relevant considering that the FLC in Mexico constitutes a key space for the promotion of healthy lifestyles and disease prevention, as well as the care of malnutrition problems, including OoO and the control of NCDs^36^. This is of particular importance, since in Mexico the predominance of a medicalized care model focused on the curative approach to diseases could help explain that nutritionists mainly perform hospital functions, such as diet prescription or food service management, limiting their incorporation into prevention, health promotion, counseling, and follow-up activities in the FLC ^15^.

Other countries in the Americas region have developed successful person-centered primary health care (PHC) models, implemented with multidisciplinary teams comprising medical staff, nursing staff, community agents (also known as health promoters), HRN, and other professionals ^29^. In contrast, in Mexico, although partial efforts have been made to incorporate these profiles into health workforces ^37^, a fragmented model persists, with a strong medicalized and organizational orientation ^38^. Likewise, studies published in Ecuador ^39^ and Mexico ^40^ indicate that these professionals are often incorporated into health services through temporary or short-term contracts, with ill-defined functions and working hours exceeding 40 hours per week. Furthermore, a study conducted in 13 Latin American countries identified the main barriers to their incorporation into health services as insufficient recognition of the profession, restricted job opportunities, weak integration into multidisciplinary teams, and the absence of specific policies ^28^. In this context, it is necessary to advance towards a federal public policy for HRH integrated into PHC, which normatively recognizes nutritionists within institutional organizational charts and allows for the reorganization of existing teams, favoring their long-term availability and sustainability within the public health system ^28^^,^^37^^,^^38^. Likewise, future research should delve into the hiring and retention mechanisms through which HRN are integrated into the Mexican health system.

However, the importance of this study lies in its provision of a national and disaggregated baseline that allows for the visualization of a scarcely documented component of the health system. The incorporation of the CI into the analysis allows for the identification of gaps in the distribution of nutrition personnel among the federal entities ^41^. Our findings show a higher concentration of HRN in states with a lower level of marginalization, and particularly, in Mexico City, which maintained the highest density during the analyzed years. This concentration can be explained, in part, by the historical centralization of health services, including human resources, and contributes to widening the existing territorial gaps in access to nutritional and specialized care in the country ^42^. This evidence is necessary to move from decisions based on historical infrastructure or hospital demand towards personnel planning oriented towards epidemiological needs, social vulnerability, and the preventive functions of primary care ^29^^,^^43^. In Mexico’s current scenario, the current availability of HRN might not be sufficient to contribute to effectively responding to the problems of Overweight and Obesity (OoO) and Non-Communicable Diseases (NCDs), which dominate the country’s epidemiological profile and whose burden is expected to increase in the coming years ^5^^,^^7^^,^^8^.

Among the main limitations of the study is the absence of a set of standardized indicators to estimate HRN density that consider differences according to the health system structure and disease burden. Also, the available sources did not allow characterizing the specific functions -clinical, administrative, food service, health promotion, teaching, or research- of the HRN, nor determining possible affiliations to more than one establishment, which could overestimate its availability. Therefore, the results represent the registered institutional availability. Furthermore, as this is an ecological study, the findings describe patterns of availability and inequality among federal entities, but do not allow inferring variability within states, particularly between urban and rural areas.

Additionally, since the CI is a relative measure, the results do not allow quantifying the absolute magnitude of personnel deficits between entities. Future studies could complement these results with estimates of HRN gaps and needs by federal entity and level of care, using reference indicators adjusted to the national context that allow more precise guidance for resource planning and allocation. Finally, the estimation of density at the second and third levels used as a denominator a proxy corresponding to 22% of the potentially user population ^(^^23^, given the absence of national information to estimate said population. Although sensitivity analyses showed that the gaps persisted under different analyzed assumptions, this parameter, derived from information for a non-Mexican population and developed three decades ago given a different epidemiological, demographic, and institutional profile, could over- or underestimate the real demand for specialized services, and therefore, the identified personnel densities.

In conclusion, despite the increase in HRN density between 2019 and 2023, significant inequalities persist in their distribution among federal entities and levels of care in Mexico. The concentration of nutritionists in second and third level units, along with lower availability in FLC, evidences a still limited incorporation of these professionals in prevention and health promotion functions. These findings show that increasing the number of HRN is not enough: it is necessary to guide their planning, distribution, and integration into health services according to the population’s health needs. Therefore, it is essential to establish reference parameters for HRN density in the Mexican context and develop public policies that favor their incorporation, equitable distribution, and availability, particularly in FLC. Strengthening the presence of nutrition professionals at this level can contribute to expanding the public health system’s capacity to respond to OoO problems and other forms of malnutrition, as well as to the prevention and control of NCDs.
