# Supplementary material for: Much obesity, scarce staff: the inequality of human resources in nutrition in Mexico (2019-2023)
Source: Rev Peru Med Exp Salud Publica. 2026 Sep 30;43(3):338–48. doi: 10.17843/rpmesp.2026.433.16389 (PMC13718575; doi:10.17843/rpmesp.2026.433.16389)
Supplement: Translation

# Translation: Mucha obesidad, poco personal: la desigualdad de los recursos humanos en nutrición en México (2019-2023)

**Authors:** Elizabeth Hoyos-Loya, Jair Yañez-Santaolalla, María Cecilia González-Robledo, David A. Barrera-Núñez, Mónica Ancira-Moreno, Karla María Armendáriz-Acosta, Hortensia Reyes-Morales, Jaqueline E. Alcalde Rabanal

**Affiliations:** 1 Centro de Investigación en Sistemas de Salud, Instituto Nacional de Salud Pública, Cuernavaca, Morelos, México.; 2 Centro de Investigación en Salud Poblacional, Instituto Nacional de Salud Pública, Cuernavaca, Morelos, México.; 3 Observatorio Materno Infantil, Departamento de Salud, Universidad Iberoamericana, Ciudad de México, México.; 4 Departamento de Ciencias de la Salud, Universidad Autónoma de Ciudad Juárez, Ciudad Juárez, Chihuahua, México.

**Keywords:** Recursos Humanos, Personal de Nutrición, Servicios de Salud, Monitoreo de las Desigualdades en Salud, Niveles de Atención de Salud, Obesidad

## Abstract

**Objetivos.:**

Analizar la densidad y desigualdad de recursos humanos en nutrición (RHN) en el sistema público de salud mexicano, así como su evolución entre 2019 y 2023.

**Materiales y métodos.:**

Se realizó un estudio ecológico con fuentes secundarias de información para 2019 y 2023. Se estimó la densidad de RHN por cada 100 000 habitantes, con base a la población total y se analizó su distribución según institución de seguridad social, nivel de atención y entidad federativa. Además, se estimó la densidad de RHN para adultos con sobrepeso u obesidad (SuO) para 2023. Para estimar la desigualdad en la distribución estatal del RHN, se utilizó el índice de concentración (IC) y como estratificador el índice de marginación.

**Resultados.:**

En México, la densidad de RHN aumentó de 5,9 en 2019 a 6,9 por cada 100 000 habitantes en 2023, sin embargo, este incremento fue desigual entre entidades federativas, instituciones y niveles de atención. En unidades de segundo y tercer nivel de atención la densidad alcanzó hasta 22,41 RHN por cada 100 000 habitantes, mientras que, en primer nivel fue de 2,0. Existe una mayor concentración de RHN en entidades con menor marginación (IC=0,13 en 2019; IC=0,11 en 2023), unidades para población con seguridad social (IC=0,36 en 2019; IC=0,33 en 2023) y de segundo y tercer nivel (IC=0,20 en 2019; IC=0,18 en 2023).

**Conclusiones.:**

En México, entre 2019 y 2023 aumentó la densidad de RHN, pero persisten desigualdades estatales y por nivel de atención. Fortalecer su planeación y distribución, particularmente en el primer nivel de atención, resulta indispensable para responder a las necesidades nutricionales y epidemiológicas del país.

## INTRODUCCIÓN

Un sistema de salud que garantice la atención a toda su población requiere contar con personal de salud capacitado y en cantidad suficiente para asegurar la prestación efectiva y de calidad de los servicios ^1^. No obstante, en la práctica la disponibilidad y distribución de los recursos humanos en salud (RHS) continúa siendo heterogénea a nivel mundial, con diferencias entre países y regiones que pueden limitar o comprometer la capacidad de respuesta de los sistemas de salud ^1^^,^^2^. A esta problemática se suma la escasez de RHS proyectada por la Organización Mundial de la Salud (OMS), donde se prevé un déficit de 11 millones de profesionales para el año 2030, que afectará particularmente a países de ingresos medios y bajos, incluido México ^3^.

Frente a este escenario, durante las últimas dos décadas, la OMS y otras organizaciones internacionales han incorporado el fortalecimiento de los RHS en la agenda global, con énfasis en su disponibilidad y accesibilidad. Entre los esfuerzos más relevantes se encuentra la Estrategia Mundial sobre Recursos Humanos para la Salud publicada en 2016, que enfatiza la necesidad de contar con RHS suficientes, adaptados a las condiciones locales y alineado con las necesidades sanitarias actuales y futuras de las poblaciones ^(^^1^.

Estas recomendaciones adquieren especial importancia en países como México, que durante las últimas décadas, ha experimentado una transformación en su perfil epidemiológico. Las distintas formas de mala nutrición -tanto por exceso como por deficiencia de nutrientes- se han consolidado como un importante problema de salud pública y desafío para el sistema de salud, destacando las elevadas prevalencias de sobrepeso u obesidad (SuO) ^4^. Según la Encuesta Nacional de Salud y Nutrición (ENSANUT) (2020-2023), 36,5% de los escolares, 40,4% de los adolescentes y 75,2% de los adultos viven con SuO ^5^^,^^6^. Además, 29,9% de la población adulta tiene un diagnóstico de hipertensión y 18,3% de diabetes, donde solo uno de cada tres adultos cuenta con control metabólico de la enfermedad ^7^^,^^8^. Las complicaciones derivadas de estas condiciones se encuentran entre las principales causas de defunción en el país ^9^.

La obesidad debe reconocerse y tratarse como una enfermedad crónica y compleja, determinada por la interacción de factores biológicos, conductuales y sociales ^10^. Su atención requiere de un modelo de atención integral e interdisciplinario, sustentado en equipos de salud multidisciplinarios -que incluyan profesionales de medicina, enfermería, psicología y nutrición- entre otros, con capacidades suficientes para el diagnóstico, tratamiento y seguimiento integral y organizado. La evidencia muestra que estos modelos pueden mejorar los resultados cardiometabólicos y la calidad de vida de las personas que viven con dicha condición ^11^^,^^12^.

En este contexto, aunque la promoción de hábitos saludables y la consejería básica pueden ser realizadas por distintos integrantes del equipo de salud, el recurso humano en nutrición (RHN) desempeña un rol fundamental, y aporta las competencias específicas para realizar la evaluación y el diagnóstico nutricional, diseñar e implementar planes individualizados de intervención, prescribir tratamiento nutricional y monitorear los resultados de las intervenciones en personas con SuO y sus comorbilidades asociadas ^13^^,^^14^. Además, el RHN participa en el diseño, implementación y evaluación de estrategias y programas de alimentación saludable en los ámbitos individual, y poblacional. Estas competencias clínicas y de salud pública distinguen la atención nutricional especializada de la consejería general y sustentan la necesidad de incorporarlos en los servicios de salud ^15^. Su integración en los servicios de salud se ha asociado con mejores resultados en salud y con el fortalecimiento de las capacidades de otros RHS para el abordaje integral de SuO así como de otras condiciones crónicas ^16^.

Sin embargo, la disponibilidad y distribución de los RHN están condicionadas por la organización y estructura de los sistemas de salud. En México, el sistema de salud es altamente segmentado y fragmentado; la población con empleo formal cuenta con acceso a las instituciones de seguridad social (Instituto Mexicano del Seguro Social -IMSS-, Instituto de Seguridad y Servicios Sociales de los Trabajadores del Estado -ISSSTE-, Petróleos Mexicanos -PEMEX-, Secretaría de la Defensa Nacional -SEDENA- y Secretaría de Marina -SEMAR-), mientras que las personas con empleo informal -incluidos desempleados, amas de casa y otros grupos poblacionales- pueden acceder a los Servicios Estatales de Salud y al Organismo Público Descentralizado Denominado Servicios de Salud del Instituto Mexicano del Seguro Social para el Bienestar (OPD-IMSS-Bienestar), que incluye atención en centros de salud hasta Institutos Nacionales, donde se brinda atención de especialidad. Cada institución dispone de lineamientos diferenciados de prestación y financiamiento, lo cual incide en la heterogeneidad del modelo de prestación de servicios y en la asignación de funciones para los RHS según la institución en la que trabajan. En consecuencia, la prioridad de contratación, disponibilidad y distribución de los RHN también difiere entre instituciones ^17^.

A pesar de la importancia de los RHN, su disponibilidad en el sistema de salud mexicano ha sido poco documentada. Por lo anterior, el objetivo de este estudio fue analizar la densidad y desigualdad de RHN en el sistema público de salud mexicano, así como su evolución entre 2019 y 2023.

**MENSAJES CLAVE**

Motivación para realizar el estudio. Los recursos humanos en nutrición (RHN) desempeñan un papel fundamental en la prevención y atención de enfermedades; sin embargo, su disponibilidad y distribución en el sistema público de salud de México han sido poco estudiadas.

Principales hallazgos. Entre 2019 y 2023, la densidad nacional de RHN aumentó de 5,97 a 6,91 por cada 100 000 habitantes. No obstante, su crecimiento fue desigual, concentrándose en entidades con menor marginación, unidades de segundo y tercer nivel y servicios dirigidos a población con seguridad social.

Implicancias en salud pública. México requiere de una política pública federal, articulada con la atención primaria de salud, que favorezca la distribución, reorganización e incorporación sostenible de RHN en el sistema público de salud.

## MATERIALES Y MÉTODOS

Se llevó a cabo un estudio ecológico, exploratorio y de grupos múltiples, con una comparación temporal entre los años 2019 y 2023.

### Fuentes de información

Se utilizaron bases de datos secundarias y registros administrativos para estimar el tamaño de los RHN, la población y adultos con SuO. Para los RHN se utilizó información de acceso abierto de la base de datos de Recursos en Salud Sectorial de la Dirección General de Información en Salud (DGIS), la cual contiene información a nivel nacional sobre las características de las unidades de salud y los recursos humanos disponibles ^18^. Se siguió un proceso de depuración que permitió identificar los registros correspondientes al universo de estudio. Los detalles por niveles de atención y tipo de institución se detallan en la sección de construcción de los numeradores.

Para el tamaño de la población a nivel nacional, se utilizó la base de datos de Población de la DGIS la cual presenta proyecciones a mitad del año del padrón poblacional por grupo etario, condición de seguridad social y entidad federativa hasta el año 2070 ^19^.

Para el cálculo de personas adultas (≥19 años) con SuO a nivel nacional y estatal, utilizamos información de la ENSANUT Continua del periodo 2020 a 2024, la cual evalúa el estado de salud y nutrición de la población mexicana y que, en su conjunto, tiene representatividad a nivel estatal ^20^.

### Densidad de RHN

Siguiendo el marco de referencia propuesto por la OMS/UNICEF para la evaluación de la disponibilidad de RHN en población general, el equipo de investigación desarrolló y operacionalizó seis de los siete indicadores utilizados en este estudio (tabla 1)^16^^,^^21^^,^^22^. Todas las densidades se estimaron a nivel nacional y estatal por cada 100 000 habitantes para el periodo 2019 a 2023, con excepción de la densidad de RHN para adultos con SuO, que se calculó únicamente para 2023. Esto se debió a las limitaciones en la representatividad estatal de las estimaciones de la población adulta con SuO para cada uno de los años del periodo 2020-2023, por lo que se utilizó la información disponible de la ENSANUT Continua 2020-2024. En 2019, no se realizó un levantamiento específico de la ENSANUT que permitiera estimar la población adulta con SuO.

**Tabla 1:** Ecuaciones para la estimación de las densidades de recursos humanos en nutrición.

RHN: recursos humanos en nutrición.

*Construcción de los numeradores*

Se incluyó como RHN a nutriólogos/as y dietistas registrados en instituciones públicas de salud que ofrecen servicios en unidades de consulta externa y hospitalarias ^16^. Se excluyó personal de nutrición que trabaja en unidades de apoyo administrativo, oficinas, laboratorios, unidades de asistencia social, centros de prevención de adicciones debido a que no brindan servicios nutricionales.

Para los RHN en unidades de salud que atienden a población sin seguridad social, se consideró al personal adscrito a unidades de los Servicios Estatales de Salud, OPD IMSS-Bienestar y programa IMSS-Bienestar. En contraste, los RHN en unidades públicas destinadas a población con seguridad social incorporaron a profesionales del IMSS, ISSSTE -incluyendo sus unidades estatales-, PEMEX, SEDENA y SEMAR. 

En cuanto al nivel de atención, los RHN se clasificaron de acuerdo con la categoría de unidad reportada en la base de datos. Se consideraron como unidades de primer nivel de atención (PNA) aquellas clasificadas como «consulta externa» que incluyen centros de salud, unidades de medicina familiar, unidades de especialidades médicas y unidades móviles. En el segundo y tercer nivel de atención, se incluyeron los profesionales adscritos a unidades clasificadas como «hospitalarias», correspondientes a hospitales generales, regionales y de especialidad, así como a los institutos nacionales de salud ^22^.

*Construcción de los denominadores*

La población de referencia correspondió a la población total estimada a mitad de año, para todas las edades, a nivel nacional y estatal. Esta misma información, también se utilizó para realizar las estimaciones según condición de seguridad social.

Por nivel de atención, se consideró que toda la población puede ser usuaria potencial del PNA dado que cualquiera puede necesitar estos servicios. Sin embargo, para la población potencial de segundo y tercer nivel, se asumió que la población que podría requerir atención hospitalaria o de especialistas en un año es de 22,0% ^23^.

Adicionalmente, para estimar la población adulta con SuO, se integró una base de datos con la información de ENSANUT Continua 2020-2024. Se agruparon estos levantamientos para tener representatividad a nivel estatal del indicador de interés ^24^. Para ello, se identificó a los participantes que contaban con datos completos de peso y talla. Con esta información se calculó el índice de masa corporal (IMC), clasificando como población con sobrepeso a quienes presentaron valores promedio de IMC entre 25,0 y 29,9 kg/m^2^, y población con obesidad quienes registraron valores iguales o superiores a 30,0 kg/m^2^. Se excluyeron valores no plausibles, definidos como IMC <10,0 y >58,0 kg/m^2 (^^5^. Para obtener el denominador correspondiente a la población adulta con SuO, se estimaron los tamaños poblacionales absolutos utilizando los factores de expansión de la ENSANUT Continua 2020-2024. Dado que se agruparon múltiples levantamientos de la encuesta, los factores de expansión originales se ajustaron dividiéndolos entre el número de levantamientos disponibles por entidad federativa (cinco ciclos para la mayoría de los estados, y cuatro para Sinaloa debido a la falta de datos en 2024). Este ajuste permitió obtener un peso ponderado anualizado. Considerando las variables de diseño de la encuesta, se estimó el total de la población anual promedio con SuO para cada estado. Para incorporar la variabilidad muestral de la encuesta, se calcularon intervalos de confianza al 95% (IC95%) para los totales poblacionales estatales. Así, el límite superior de la densidad se calculó utilizando el límite inferior del intervalo de confianza del tamaño poblacional como denominador, mientras que el límite inferior de la densidad se estimó utilizando el límite superior del intervalo de confianza del tamaño poblacional.

Para evaluar los cambios en las densidades entre 2019 y 2023, se calculó el porcentaje de cambio entre ambos años. Asimismo, se utilizó una prueba t de Student de dos colas para evaluar la significancia estadística de las diferencias observadas. Se consideró estadísticamente significativo un valor de p<0,05.

*Análisis de desigualdad*

Para cuantificar la desigualdad en la distribución de RHN entre instituciones para población con y sin seguridad social, así como entre niveles de atención y entidad federativa, se utilizaron estimadores de razón de densidad. Esta métrica permite comparar la frecuencia relativa de los indicadores, proporcionando una medida simple de las diferencias en su distribución ^25^.

La desigualdad en la distribución de RHN entre las 32 entidades federativas se estimó mediante el índice de concentración (IC) ^26^, utilizando el índice de marginación (IM) estatal, estimado por el Consejo Nacional de Población (CONAPO), como estratificador de equidad. El IM es un indicador multidimensional que evidencia las inequidades socioespaciales presentes entre los estados del país. Su cálculo se basa en nueve indicadores agrupados en cuatro dimensiones: educación, condiciones de la vivienda, ingresos monetarios y distribución poblacional. Debido a que este índice se estima cada cinco años, para el presente análisis se utilizó el IM de 2020 ^27^. La fórmula para estimar el IC es la siguiente ^26^:



El resultado del IC oscila entre -1 y 1. Un IC negativo indica que la densidad de RHN se concentra entre los estados más marginados (IM muy altos). Mientras que, IC positivos, indican que la densidad se concentra en los estados menos marginados (IM muy bajo). Si el IC toma el valor de cero, indica ausencia de desigualdad ^26^. Para saber si las diferencias del IC entre ambos años son estadísticamente significativas, realizamos una prueba t de Student de dos colas para cada indicador. Se consideraron valores p<0,05 como significativos.

Se generaron las curvas de concentración para cada uno de los indicadores. La cual muestra la representación gráfica de la desigualdad, al relacionar la dimensión socioeconómica -IM- (eje X) con el indicador de recursos en salud -RHN- (eje Y), en ausencia total de desigualdad la curva toma la forma de una línea recta con 45 grados de inclinación, a la que se le llama línea de igualdad (IC=0). El área por debajo o superior a esta línea de 45 grados corresponde al valor del IC. El procesamiento de los datos se realizó utilizando el paquete estadístico Stata versión.17.0 (Stata Statistical Software, 2017; StataCorp, College Station,TX).

## RESULTADOS

Entre 2019 y 2023, la densidad nacional de RHN aumentó de 5,97 a 6,91 por cada 100 000 habitantes, lo que representa un incremento en el periodo de 15,75%. Mientras que, para población con SuO en 2023, la densidad nacional alcanzó 14,09 RHN por cada 100 000 adultos con ambas condiciones (IC 95% 13,66‒14,54). En cuanto a los RHN en instituciones para población con y sin seguridad social, las densidades resultaron similares, sin embargo, el mayor incremento se registró en instituciones para población con seguridad social, con un aumento de 25,8% (p<0,05) al pasar de 5,62 RHN en 2019 a 7,07 en 2023; mientras que, en instituciones para población sin seguridad social, hubo un incremento de 8,13% al pasar de 6,27 en 2019 a 6,78 en 2023.

Al analizar por nivel de atención, la mayor concentración de recursos se observó en las unidades de segundo y tercer nivel, con 20,09 y 22,41 RHN por cada 100 000 habitantes en 2019 y 2023, respectivamente. Sin embargo, el mayor incremento por nivel de atención, se registra en unidades de PNA, con 27,74% (p<0,05). Sin embargo, estas unidades presentaron las tasas más bajas de RHN: 1,55 en 2019 y 1,98 en 2023. La razón de densidad entre el PNA y las unidades de segundo y tercer nivel pasó de 12,96 en 2019 a 11,31 en 2023, lo que equivale a un RHN en PNA por cada 11 en unidades de segundo y tercer nivel (tabla 2).

**Tabla 2:** Cambios en la disponibilidad de recursos humanos en nutrición por cada 100 000 habitantes en México, 2019 y 2023.

Categoría		2019			2023			% de cambio	Valor de p^a^
		n	Densidad	Razón de densidad	n	Densidad	Razón de densidad		
Nacional		7569	5,97	ND	9070	6,91	ND	15,75	<0,001
Nacional en adultos con sobrepeso y obesidad		ND	ND	ND	9070	14,09 (13,66-14,54)^b^	ND	ND	ND
Seguridad social									
	Instituciones para población sin seguridad social	4333	6,27	Ref.^a^	4825	6,78	Ref.	8,13	0,140
	Instituciones para población con seguridad social	3236	5,62	0,90	4245	7,07	1,04	25,80	<0,001
Por nivel de atención									
	Unidades de primer nivel	1974	1,55	Ref.	2599	1,98	Ref.	27,74	0,021
	Unidades de segundo y tercer nivel	5595	20,09	12,96	6471	22,41	11,31	11,54	<0,001
Población total		126 577 891			131 230 255			3,67	

a Prueba t de Student de dos colas. Se consideró estadísticamente significativo un valor p<0,05, ^b^ intervalo de confianza al 95%.

Ref: referencia para la estimación de la razón de la densidad, ND: información no disponible.

Al desagregar por entidad federativa, en 2023, el 40,62% de las entidades (n=13) presentó densidades superiores a la media nacional (6,91 por cada 100 000 habitantes). Las densidades más altas se observaron en la Ciudad de México (15,58), Tabasco (13,61) y Yucatán (11,19), mientras que las menores corresponden a los estados de Puebla (4,42), Oaxaca (4,02) y San Luis Potosí (3,59). El análisis del porcentaje de cambio a lo largo del periodo evidencia una variabilidad considerable entre las entidades federativas; 65,62% de las entidades (n=21) registró incrementos superiores al promedio nacional (15,75%), con aumentos que alcanzaron hasta 89,22% en Hidalgo y 75,44% en Sinaloa. Por el contrario, 15,62% de las entidades (n=5) mostró reducciones en la densidad de RHN, destacando Tlaxcala, con una disminución del 33,91% y el Estado de México, con 21,15% (figura 1).

**Figura 1:** Densidad y porcentaje de cambio en la tasa de recurso humano en nutrición por cada 100 000 habitantes en México, 2019 y 2023.

^a^Prueba t de Student de dos colas. Se consideró estadísticamente significativo un valor de p<0,05. Para la Ciudad de México (CDMX) el valor de p<0,001.

El IC reveló que la mayor densidad de RHN se concentra en entidades con menores grados de marginación (IM: muy bajo), con valores positivos y estadísticamente significativos para los años 2019 y 2023 (IC= 0,13 en 2019, p<0,05; IC=0,11 en 2023, p<0,05). Al desagregar el IC por tipo de institución pública de salud, se observó una mayor desigualdad, estadísticamente significativa, en la distribución estatal de RHN en las instituciones que atienden a población con seguridad social (IC=0,36 en 2019, p<0,05; IC=0,33 en 2023, p<0,05), en comparación con las instituciones que atienden a población sin seguridad social. Al analizar el IC por nivel de atención, la desigualdad fue más pronunciada en unidades de segundo y tercer nivel (IC=0,20 en 2019, p<0,05; IC=0,18 en 2023, p<0,05). En las unidades de PNA, el IC presentó valores negativos y cercanos a cero en 2019 (IC=−0,05) y 2023 (IC=−0,02) (tabla 3). Si bien, los porcentajes de cambio indican reducciones del IC entre 2019 y 2023 en los indicadores, estas diferencias no resultaron ser estadísticamente significativas.

**Tabla 3:** Índice de concentración del recurso humano en nutrición según grado de marginación estatal. México, 2019 y 2023.

		2019			2023			% de cambio	Valor de p^b^
		IC	Valor de p^a^	EE	IC	Valor de p^a^	EE		
Nacional		0,13	<0,001	0,04	0,11	<0,001	0,04	-15,38	0,725
Adultos con sobrepeso u obesidad		ND	ND	ND	0,06	0,076	0,05	ND	ND
	Seguridad social								
	Instituciones para población sin seguridad social	0,14	0,073	0,07	0,08	0,161	0,06	-42,86	0,518
	Instituciones para población con seguridad social	0,36	<0,001	0,07	0,33	<0,001	0,07	-8,33	0,763
Por nivel de atención									
	Unidades de Primer nivel	-0,05	0,352	0,05	-0,02	0,553	0,04	-60,00	0,641
	Unidades de Segundo y tercer nivel	0,20	<0,001	0,04	0,18	<0,001	0,04	-10,00	0,725

N=32

a Prueba de Wald para el IC. Se consideró estadísticamente significativo un valor de p<0,05.

b Prueba t de Student de dos colas. Se consideró estadísticamente significativo un valor de p<0,05.

IC: índice de concentración, EE: error estándar, ND: información no disponible.

Las curvas de concentración para la densidad de RHN a nivel nacional, en adultos con SuO, por tipo de seguridad social, así como por nivel de atención, fueron consistentes con los resultados del IC, al ubicarse por debajo de la línea de equidad, evidenciando una mayor concentración de RHN en entidades con menor marginación. Por otra parte, la curva correspondiente a las unidades de PNA debe interpretarse con cautela, debido a que presenta cruces con la línea de equidad, lo que podría reflejar la coexistencia de desigualdades de magnitud y dirección opuestas a lo largo de la distribución de la marginación (figura 2).

**Figura 2:** Curvas de concentración del recurso humano en nutrición según grado de marginación estatal. México, 2019 y 2023.

N=32 RHN: recursos humanos en nutrición

## DISCUSIÓN

Hasta donde sabemos, este es el primer estudio, que documenta la disponibilidad y distribución de los RHN en las instituciones públicas de salud de México. Entre 2019 y 2023, la densidad nacional de estos profesionales aumentó de 5,97 a 6,91 por cada 100 000 habitantes. Sin embargo, esta expansión fue desigual entre entidades federativas, instituciones y niveles de atención. Los RHN continuaron concentrándose en las instituciones de seguridad social y unidades de segundo y tercer nivel de atención (22,41 RHN por cada 100 000 habitantes en 2023), mientras que las unidades de PNA registraron una densidad de 1,98 profesionales por cada 100 000 habitantes durante el mismo año. Los IC positivos muestran que los RHN se ubicaron principalmente en entidades con menor grado de marginación. Esta distribución podría limitar la capacidad del sistema para realizar acciones de prevención, detección oportuna y atención integral para múltiples problemas relacionados con el SuO, así como de enfermedades crónicas no transmisibles (ECNT) en contextos ambulatorios.

El incremento moderado de la densidad nacional de RHN no debe interpretarse, por tanto, como evidencia de que México haya resuelto su brecha de fuerza laboral. En primer lugar, no existe una norma nacional o internacional que establezca una densidad mínima o de referencia de RHN necesaria para responder a las necesidades de la población (carga de la enfermedad y el tamaño poblacional), lo que dificulta cuantificar las brechas. Por ello, es necesario establecer un mínimo indispensable o parámetros de planeación contextualizados que permita garantizar una cobertura adecuada, considerando que su función va más allá de la prescripción de dietas en el ámbito hospitalario y que su participación es particularmente relevante en el PNA para promover una nutrición saludable. En segundo lugar, el aumento observado de RHN ha estado marcado por la contratación temporal y su incorporación a programas específicos, más que una estrategia sostenida de planeación de RHS ^28^. Finalmente, esta densidad debe interpretarse a la luz del contexto epidemiológico, caracterizado por una elevada prevalencia de SuO desde etapas tempranas de la vida, así como de diabetes e hipertensión que continúan presentando un control insuficiente ^(^^7^^,^^8^.

Nuestros hallazgos son consistentes con la evidencia de otros sistemas de salud en América Latina. En Brasil, el incremento cuantitativo de estos profesionales no ha resuelto por sí solo las disparidades geográficas ^29^, mientras que en nuestro estudio se observa una concentración similar de RHN en regiones de menor marginación. Asimismo, existe una asimetría crítica por niveles de atención. Mientras que en el Perú el 7,93% de los establecimientos de PNA cuenta con un nutriólogo/a ^30^, en México, en 2023 había un profesional de nutrición en el PNA por cada 11 ubicados en unidades de mayor complejidad. A diferencia de Perú ^30^^,^^31^, nuestro estudio incorpora dos dimensiones no exploradas en el análisis peruano: la distribución según condición de seguridad social y la estimación formal de las desigualdades mediante el IC estatal ^30^.

La evidencia es sólida al señalar los múltiples beneficios a la salud poblacional y a los sistemas de salud al integrar este recurso en sus equipos de atención ^32^^-^^34^. Estos beneficios van desde la mejora en los indicadores clínicos ^33^, como por ejemplo perfil lipídico, glucémico, IMC y presión arterial hasta la reducción de costos sanitarios mediante una menor utilización de servicios de salud que contribuye a disminuir también la saturación de servicios hospitalarios y gasto sanitario ^32^^-^^34^. En conjunto, estos hallazgos demuestran que invertir en RHN, principalmente de atención primaria, podría constituir una estrategia costo-efectiva para fortalecer la respuesta sanitaria ^32^^-^^35^.

Por otra parte, el estudio también identifica una marcada brecha en la distribución de RHN entre los hospitales de segundo y tercer nivel y las unidades de PNA, consistente con lo observado en Brasil y Perú ^29^^-^^31^. Esta concentración resulta relevante considerando que el PNA en México constituye un espacio clave para la promoción de estilos de vida saludables y prevención de enfermedades, así como la atención de problemas de mala nutrición, incluido el SuO y el control de las ECNT ^36^. Esto es de particular importancia, ya que en México el predominio de un modelo de atención medicalizado y centrado en el abordaje curativo de las enfermedades, podría contribuir a explicar que los nutriólogos/as desempeñen principalmente funciones hospitalarias, como la prescripción de dietas o la administración de servicios de alimentos, limitando su incorporación en actividades de prevención, promoción de la salud, consejería y seguimiento en el PNA ^15^.

Otros países de la región de las Américas han desarrollado modelos exitosos de atención primaria en salud (APS) centrados en las personas, implementados con equipos multidisciplinarios integrados por personal médico, de enfermería, agentes comunitarios (también conocidos como promotores de salud), RHN y otros profesionales ^29^. En contraste, en México, si bien se han realizado esfuerzos parciales para incorporar estos perfiles en las plantillas de salud ^37^, persiste un modelo fragmentado, con una fuerte orientación medicalizada y organizacional ^38^. Asimismo, estudios publicados en Ecuador ^39^ y México ^40^ señalan que estos profesionales suelen incorporarse a los servicios de salud mediante contratos temporales o de corto plazo, con funciones poco definidas, y jornadas laborales superiores a las 40 horas semanales. Por otra parte, un estudio realizado en 13 países de América Latina identificó como principales barreras para su incorporación en los servicios de salud el reconocimiento insuficiente de la profesión, las oportunidades laborales restringidas, la débil integración en equipos multidisciplinarios y la ausencia de políticas específicas ^28^. En este contexto, resulta necesario avanzar hacia una política pública federal de RHS integrada a la APS, que reconozca normativamente a los nutriólogos/as dentro de los organigramas institucionales y permita reorganizar los equipos existentes, favoreciendo su disponibilidad y sostenibilidad a largo plazo dentro del sistema público de salud ^28^^,^^37^^,^^38^. Asimismo, futuras investigaciones deberían profundizar en los mecanismos de contratación y retención mediante los cuales los RHN se integran al sistema de salud mexicano.

No obstante, la importancia de este estudio radica en que ofrece una línea basal nacional y desagregada que permite visibilizar un componente del sistema de salud escasamente documentado. La incorporación del IC al análisis permite identificar brechas en la distribución del personal de nutrición entre las entidades federativas ^41^. Nuestros hallazgos muestran una mayor concentración de RHN en estados con menor nivel de marginación, y particularmente, en la Ciudad de México, que mantuvo la mayor densidad durante los años analizados. Esta concentración puede explicarse, en parte, por la centralización histórica de los servicios de salud, incluidos los recursos humanos, y contribuye a profundizar las brechas territoriales existentes en el acceso a la atención nutricional y especializada en el país ^42^. Esta evidencia es necesaria para avanzar de decisiones basadas en infraestructura histórica o demanda hospitalaria hacia una planeación del personal orientada a las necesidades epidemiológicas, la vulnerabilidad social y las funciones preventivas de la atención primaria ^29^^,^^43^. En el escenario actual de México, la disponibilidad actual de RHN podría no ser suficiente para contribuir a responder de manera efectiva a los problemas de SuO y ECNT, que dominan el perfil epidemiológico del país y cuya carga se espera incrementen en los próximos años ^5^^,^^7^^,^^8^.

Entre las principales limitaciones del estudio se encuentra la ausencia de un conjunto de indicadores estandarizados para estimar la densidad de RHN que consideren las diferencias según la estructura del sistema de salud y la carga de la enfermedad. Asimismo, las fuentes disponibles no permitieron caracterizar las funciones específicas -clínicas, administrativas, de servicio de alimentación, promoción de la salud, docencia o investigación- del RHN, ni determinar posibles adscripciones a más de un establecimiento, lo que podría sobreestimar su disponibilidad. Por lo tanto, los resultados representan la disponibilidad institucional registrada. Además, al tratarse de un estudio ecológico, los hallazgos describen patrones de disponibilidad y desigualdad entre entidades federativas, pero no permiten inferir en la variabilidad al interior de los estados, particularmente entre zonas urbanas y rurales.

Adicionalmente, dado que el IC es una medida relativa, los resultados no permiten cuantificar la magnitud absoluta de los déficits de personal entre entidades. Futuros estudios podrían complementar estos resultados con estimaciones de brechas y necesidades de RHN por entidad federativa y nivel de atención, utilizando indicadores de referencia ajustados al contexto nacional que permitan orientar de manera más precisa la planificación y asignación de recursos. Finalmente, la estimación de la densidad en segundo y tercer nivel utilizó como denominador un proxy correspondiente al 22% de la población potencialmente usuaria ^(^^23^, ante la ausencia de información nacional para estimar dicha población. Aunque los análisis de sensibilidad mostraron que las brechas persistían bajo distintos supuestos analizados, este parámetro, derivado de información para población no mexicana y desarrollado tres décadas atrás ante otro perfil epidemiológico, demográfico e institucional, podría sobreestimar o subestimar la demanda real de servicios especializados, y por ende, las densidades de personal identificadas.

En conclusión, a pesar del incremento en la densidad de RHN entre 2019 y 2023 persisten importantes desigualdades en su distribución entre las entidades federativas y los niveles de atención en México. La concentración de nutriólogos/as en unidades de segundo y tercer nivel, junto con una menor disponibilidad en PNA, evidencia una incorporación aún limitada de estos profesionales en funciones de prevención y promoción de la salud. Estos hallazgos muestran que incrementar el número de RHN no es suficiente: es necesario orientar su planificación, distribución e integración en los servicios de salud de acuerdo con las necesidades de salud de la población. Por ello, resulta indispensable establecer parámetros de referencia para la densidad de RHN en el contexto mexicano y desarrollar políticas públicas que favorezcan su incorporación, distribución equitativa y disponibilidad, particularmente en el PNA. Fortalecer la presencia de profesionales de nutrición en este nivel puede contribuir a ampliar la capacidad del sistema público de salud para responder a los problemas de SuO y otras formas de mala nutrición, así como a la prevención y control de las ECNT.
